# Autotetraploid cell Line induced by SP600125 from crucian carp and its developmental potentiality

**DOI:** 10.1038/srep21814

**Published:** 2016-02-22

**Authors:** Yonghua Zhou, Mei Wang, Minggui Jiang, Liangyue Peng, Cong Wan, Jinhui Liu, Wenbin Liu, Rurong Zhao, Xiaoyang Zhao, Wei Hu, Shaojun Liu, Yamei Xiao

**Affiliations:** 1 Key Lab of Protein Chemistry and Developmental Biology of Education Ministry of China, College of Life Sciences, Hunan Normal University, Changsha, 410081, China; 2 State Key Laboratory of Reproductive Biology, Institute of Zoology, Chinese Academy of Sciences, Beijing, 100101, China; 3 The National Key Laboratory of Freshwater Ecology and Biotechnology, Institute of Hydrobiology, Chinese Academy of Sciences, Wuhan, 430072, China

## Abstract

Polyploidy has many advantages over diploidy, such as rapid growth, sterility, and disease resistance, and has been extensively applied in agriculture and aquaculture. Though generation of new polyploids via polyploidization has been achieved in plants by different ways, it is comparatively rare in animals. In this article, by a chemical compound, SP600125, polyploidization is induced in fish cells *in vitro*, and a stable autotetraploid cell line has been generated from diploid fibroblast cells of crucian carp. As a c-Jun N-terminal kinase (Jnk) inhibitor, SP600125 does not function during the induction process of polyploidization. Instead, the p53 signal pathway might be involved. Using the SP600125-induced tetraploid cells and eggs of crucian carp as the donors and recipients, respectively, nuclear transplantation was conducted such that tetraploid embryos were obtained. It suggests that combining polyploidization and the somatic cell nuclear transfer technique (SCNT) is an efficient way to generate polyploidy, and the presented method in this research for generating the tetraploid fish from diploid fish can provide a useful platform for polyploid breeding.

Polyploidy is useful in breeding new plant and animal species. Because it has more than two sets of chromosomes, the polyploid has some advantages over the diploid, such as rapid growth, sterility, and disease resistance. In animals, polyploids rarely occur in the wild^1^, but studies have shown that polyploidization exists in historical vertebrate evolutionary events, and it experiences for at least two rounds of the tetraploid process^2^. Allotetraploid crucian carp have been generated by distant hybridization, it is regarded as the first natural case of an allotetraploid vertebrate animal with stable genetic characters^3^.

In agriculture and fisheries, several induction methods have been reported to generate polyploidy. Temperature alternation or chemical compound treatment are often applied to polyploid generation^2^, and the so-called physical induction approach seems the most popular^4^,^5^. However, due to low efficiency, high incidence of chimera formation, and polyploid instability, these polyploidization methods can’t meet the demand of large-scale production^2^.

The somatic cell nuclear transfer technique (SCNT) has been wildly used to generate cloned animals, including fish. A new method combining the polyploid cell generation and SCNT to generate stable polyploid animals seems promising^6^,^7^,^8^,^9^,^10^. The critical step in the process is polyploid cell generation. Fortunately, new chemical compounds that induce artificial polyploidy in mammalian cells have been screened^5^, though the generation of polyploid cells in fish remains difficult. Currently, no stable polyploid fish cell lines generated by chemical induction have been reported.

Previous results indicate that the small compound SP600125 could induce polyploidization of mammalian cancer cells^11^,^12^. As a c-Jun N-terminal kinase (Jnk) inhibitor, it is reported that SP600125 can lead to G2/M phase arrest such that G2-to-M phase transition is prevented, and DNA endoreplication occurs directly from the G2 phase^11^,^13^,^14^. However, contradictory results have also suggested that SP600125 may not serve as a Jnk inhibitor to induce polyploidization^11^.

Since endoreduplication is related to polyploidization^3^,^15^,^16^, in this article, we intend to study a new polyploidization method for the autotetraploid fish by means of SP600125-induced. Specifically, a stable tetraploid crucian carp cell line from diploid fish will be first generated by a sequence of SP600125 treatments and fluorescence activated cell sorting (FACS) purification. Then, by the SCNT, the tetraploid embryos will be prepared. It is found that as a Jnk inhibitor, SP600125 does not function during the induction process of polyploidization. Instead, the p53 signal pathway seems to be involved.

## Results

### SP600125-induced Polyploidization from Diploid Crucian Carp Cells

**Figure 1:**
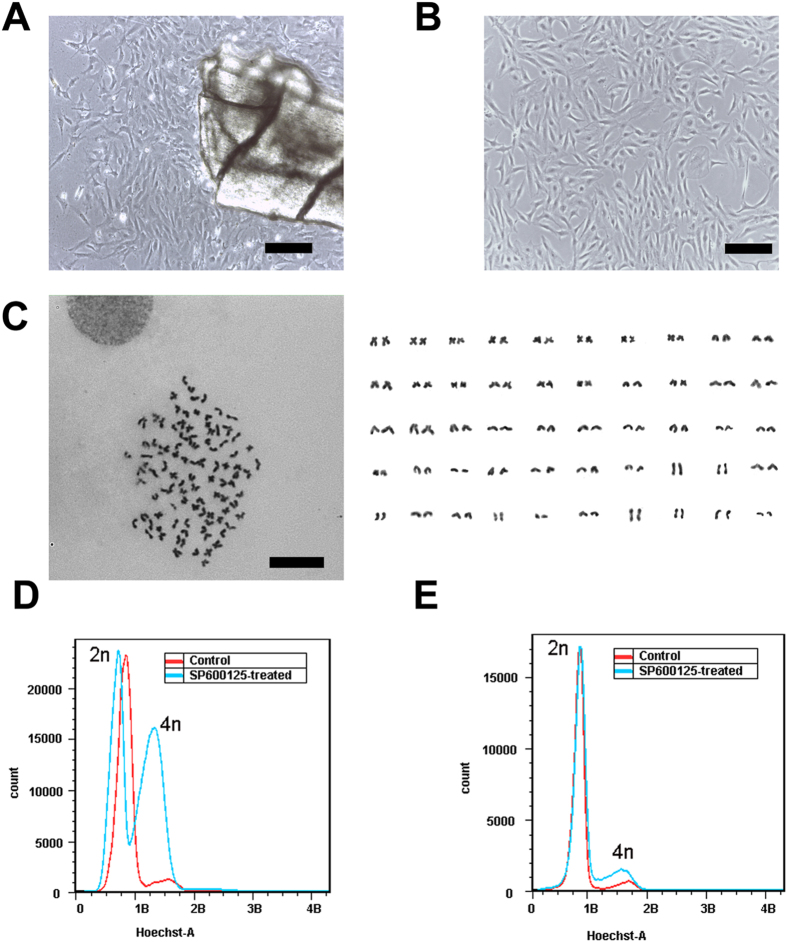
SP600125-induced polyploidization of crucian carp cells *in vitro.* (**A**) Morphology of the crucian carp cells (passage 0) *in vitro*, scale bars = 200 μm. (**B**) Morphology of the crucian carp cells (passage 5) *in vitro*, scale bars = 200 μm. (**C**) Karyotype analysis of the crucian carp cells (passage 26), 2N = 100. Scale bars = 20 μm). (**D**) FACS analysis of SP600125 (blue) or DMSO (red) treated cell DNA content. (**E**) FACS analysis of DNA content of cells collected from 4n and 8n peaks (cultured for another two passage without SP600125 (blue)), diploid crucian carp cells were used as the control (red).

To obtain tetraploid crucian carp cells, we first generated diploid crucian carp fibroblast cells. Pieces of fin were sampled from normal crucian carp and cultured. Two days later, the fin cells migrated out and formed a monolayer (Fig. 1A). The fin cells showed robust proliferative capacity, and could passage every 2 ~ 3 days (Fig. 1B). The fin cells have now been maintained for more than 40 passages. Karyotype analysis showed that the majority of cells were normal diploid (90%, 2N = 100) (Fig. 1C). These results indicate that the fin cell line was a stable diploid crucian carp cell line.

We then used SP600125 to treat the diploid carp cells and found that the cell number was obviously less than the control (Supplementary Fig. S1A); DMSO (a SP600125 solvent) was used as a negative control. FACS data showed that the tetraploid (4n) peak cells increased significantly in SP600125-treated cells compared with the control (Fig. 1D). The 4n peak cells were collected, and cultured for another 2 passages without SP600125, the proportion of the 4n peak cells decreased (less than 15%), but was slightly higher than the normal diploid cells (Fig. 1E). The 4n peak cells were growth-arrested and apoptotic after 72 h of SP600125 continuous treatment (Supplementary Fig. S1B). These results indicate that SP600125 could induce polyploidization in diploid crucian carp cells, and maintain the tetraploid state.

### Generation of Stable Autotetraploid Crucian Carp Cell Line

**Figure 2:**
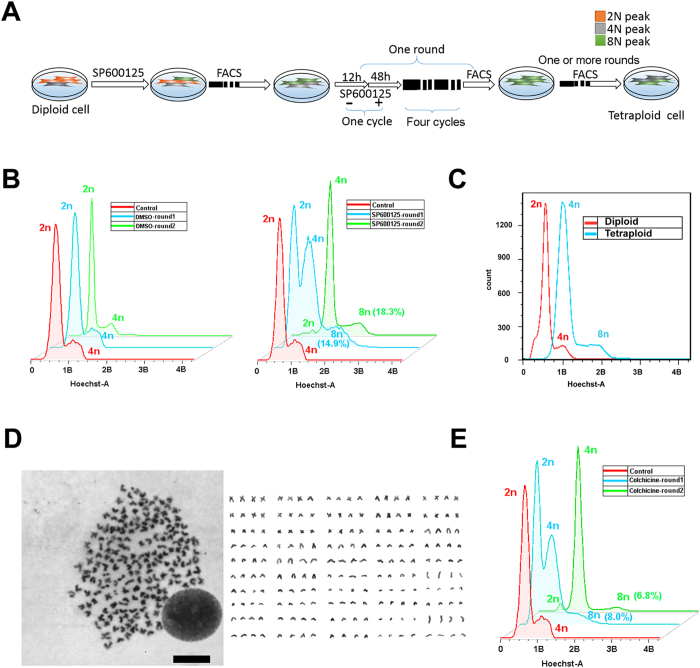
Generation of a stable tetraploid crucian carp cell line. (**A**) Scheme for tetraploid crucian carp cells generation by SP600125. (**B**) FACS analysis of crucian carp cell DNA content after one (blue) or two (green) rounds of DMSO (left) and SP600125 (right) treated, diploid crucian carp cells were used as control. (**C**) FACS analysis of tetraploid crucian carp cells generated (blue) and diploid crucian carp cell (red) DNA content. (**D**) Karyotype of tetraploid crucian carp cells (4N = 200), scale bars = 20 μm. (**E**) FACS analysis of crucian carp cell DNA content after one (blue) or two (green) rounds of Colchicine treated, diploid crucian carp cells were used as control.

To establish stable tetraploid cell line, we modified the polyploidization protocol from diploid crucian carp cells (Fig. 2A). To avoid cell death, 4n and 8n peak SP600125-treated cells were collected and cultured again in an SP600125-free medium for 12 h until the cells adhered to the dish (Supplementary Fig. S2A), they were then treated for another 48 h in a medium with SP600125. In total, we performed a 48 h SP600125 treatment in every 72 h of a passage, which was defined as one SP600125 treatment cycle. After five cycles of SP600125 treatment in five passages (also called one round, Supplementary Fig. S2B), the percentage of 8n peak cells in the SP600125-treated group was significantly higher than the control group. We then collected the 8n peak cells from the SP600125-treated cells, and performed another one or more rounds (Fig. 2B). Finally, stable tetraploid cells were generated. The FACS analysis data showed that it was a typical tetraploid cell line (Fig. 2C). Karyotype analysis revealed that the tetraploid crucian carp cells contained 200 chromosomes (Fig. 2D).

To test if this protocol could generate tetraploid cells with other chemical compounds, we selected colchicine (a drug that is always used for cell metaphase arrest) to treat the diploid crucian carp cells. Similar to SP600125, the cells exhibited severe cell death in the continuously treated group, and stable tetraploid cells were generated after two rounds of colchicine treatment. However, there were always existing small 2n peak cells, so SP600125 may be better than that of Colchicine (Fig. 2E). All of these data indicate that we were able to robustly generate tetraploid cells with this method.

### Characteristics of SP600125-induced Autotetraploid Cells

**Figure 3:**
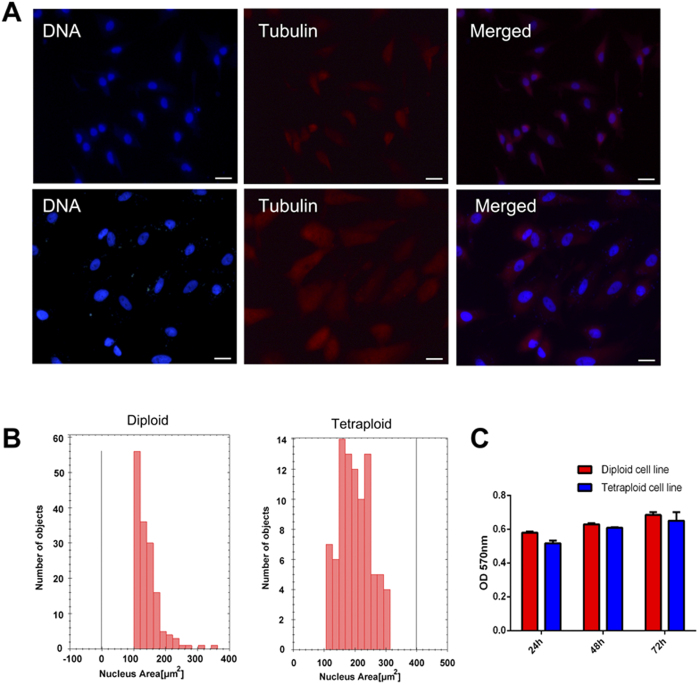
Tetraploid cell characteristics. (**A**) Tubulin (red) was detected by Immunostaining of diploid (up) and tetraploid (down). DNA (blue) was stained with Hoechst 33342. Scale bars represent 50 μm. (**B**) Nuclear size of diploid (left) and tetraploid (right) cells detected by high content assays. (**C**) MTT analysis of diploid (red) and tetraploid cells (blue). Error bars represent the means ± SD; n = 3.t = 3.186, P > 0.05.

The immune staining data revealed that the autotetraploid cell spindle bodies and nuclei were relatively larger than those of the diploid control (Fig. 3A), which might have resulted from more chromosomes contained in the tetraploid cells. We performed the nucleus area assay by High Content Screening (Supplementary Fig. S3A). Although the tetraploid cell nuclei roundness were similar in shape to the diploid carp cells (Supplementary Fig. S3B), we found that the area of the majority of tetraploid cell nuclei were between 100 ~ 300 μm^2^, while it was always about 100 μm^2^ in the diploid control cells (Fig. 3B).

We also performed the MTT assay to evaluate the growth rate of the tetraploid cell line generated. The results revealed that the proliferation of the tetraploid cells was similar to that of the diploid cells (Fig. 3C). Thus, although the tetraploid cells had a higher DNA content, the cell cycle was not affected.

### SP600125-induced polyploidization without Involvement of Jnk

**Figure 4:**
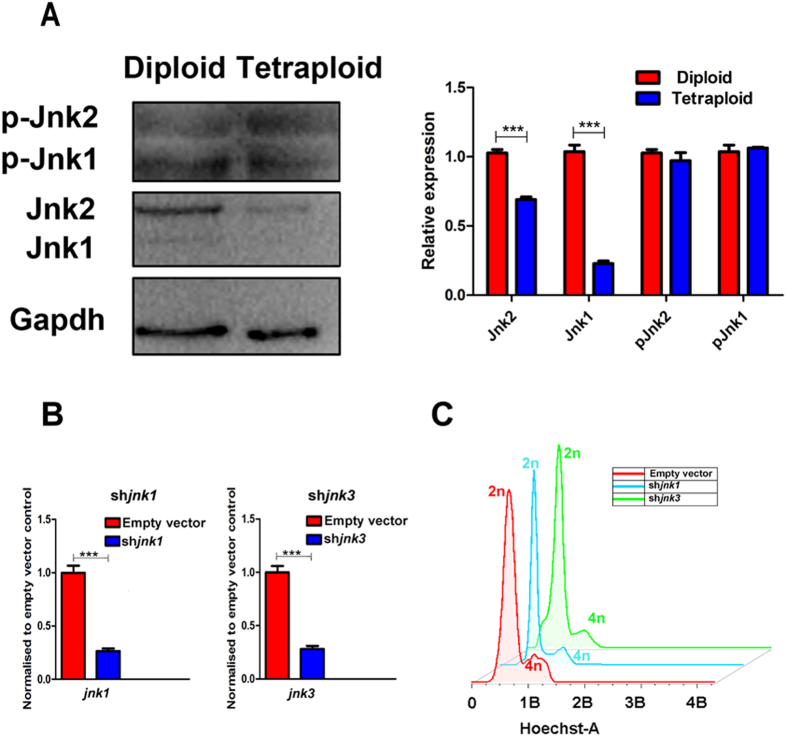
Jnk pathway did not involve in cell polyploidization. (**A**) Western blot analysis detected Jnk (Jnk1/Jnk2) expression in SP600125-induced tetraploid cells and normal diploid cells. Error bars represent the means ± SD; n = 3. P < 0.001. (**B**) shRNA knockdown of *jnk1/jnk3*. Cells transfected with an empty vector were used as control. Knockdown was evaluated by Q-PCR. Expression values are relative to actin expression on set as 1. Error bars represent the means ± SD; n = 3. P < 0.001. (**C**) FACS analysis of *jnk1*-shRNA (blue) and *jnk3*-shRNA (green) transfected cell DNA content. An empty vector (red) was transfected as the control.

To explore the mechanism underlying SP600125 in the induction of polyploidization, we first examined Jnk expression in SP600125-induced crucian carp cells by western blot, because SP600125 is a selective ATP-competitive Jnk inhibitor^17^. We found that Jnk levels were higher in the normal diploid cells than in tetraploid cells, especially Jnk1. However, the pJnk expression level, a type of activation/phosphorylation Jnk, did not differ between the tetraploid and normal diploid cells (Fig. 4A).

We further examined whether or not the *jnk1* gene was involved in cell polyploidization. Two shRNAs (sh*jnk1* and sh*jnk3*) were used to specifically knock-down *jnk1/jnk3* (Fig. 4B). After puromycin treatment, puromycin resistant cells were analyzed by FACS. The results indicated that there were no obvious differences in cell cycle between the *jnk1/jnk3* knockdown cells and the control ones, which had only been transfected with an empty shRNA vector (Fig. 4C). Additionally, there were no obvious 8n cells in either the sh*jnk1* or sh*jnk3* group (Fig. 4C). Thus, SP600125 might not function as a Jnk inhibitor during SP600125-induced polyploidization, there is likely be some other polyploidization-inducing signal pathway that is affected by SP600125 treatment.

### Involvement of p53 Signal Pathway in SP600125-induced Polyploidization

**Figure 5:**
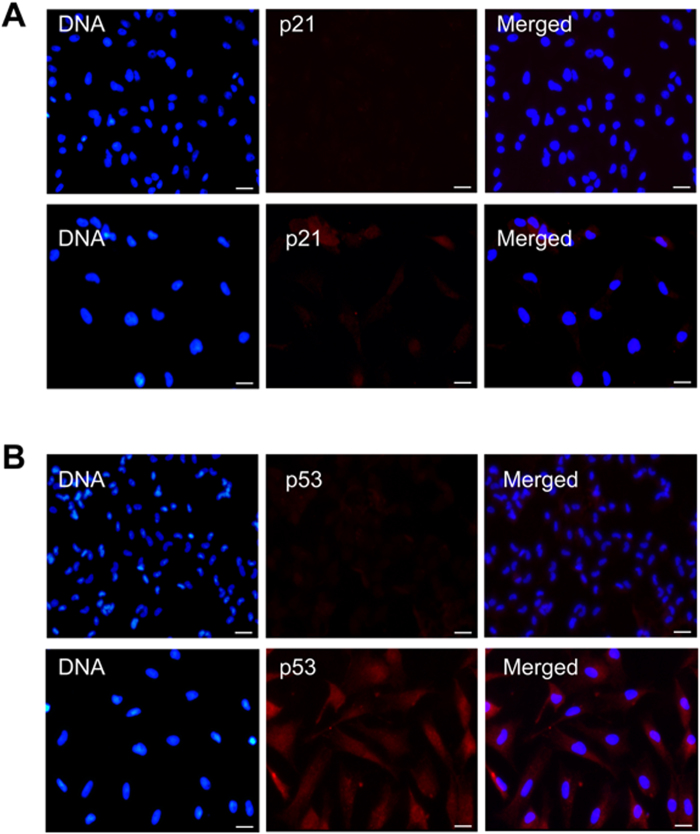
p21 and p53 expressions in tetraploid and diploid cells. (**A**) Immunostaining of p21 (red) in diploid cells (up) and tetraploid cells (down), DNA was stained with Hoechst 33342 (blue). Scale bars represent 50 μm. (**B**) Immunostaining of p53 (red) in in diploid cells (up) and tetraploid cells(down), DNA was stained with Hoechst 33342 (blue). Scale bars represent 50 μm.

Previous studies have reported a p53-dependent induction of p21Cip1/Waf1 expression during cell cycle arrest^18^. Because we detected cell cycle arrest during polyploidization, we explored their expressions in both the tetraploid and normal diploid cells. Immunofluorescence analysis revealed that both p21 (Fig. 5A) and p53 (Fig. 5B) were expressed in tetraploid but not diploid cells, and the fluorescence intensity is shown in Supplementary (Supplementary Fig. S4). Thus, the p53 signal pathway might play a role in SP600125-induced polyploidization.

### Development of the SCNT embryos

**Figure 6:**
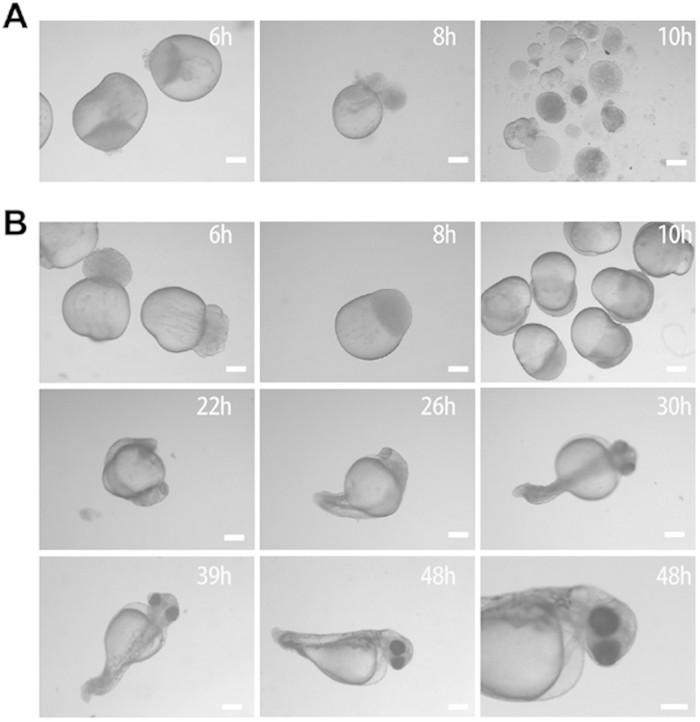
Nuclear transfer embryos derived from the SP600125-induced tetraploid cells. (**A**) reprensents the control, where all unfertilized crucian carp eggs without SCNT are died before multicellular stage. (**B**) indicates the SCNT embryos, which are the reconstructed embryos from the SP600125-induced autotetraploid cell nuclei and crucian carp eggs developing to blastula (6 h), to gastrula (10 h), even to larva (48 h).

**Figure 7:**
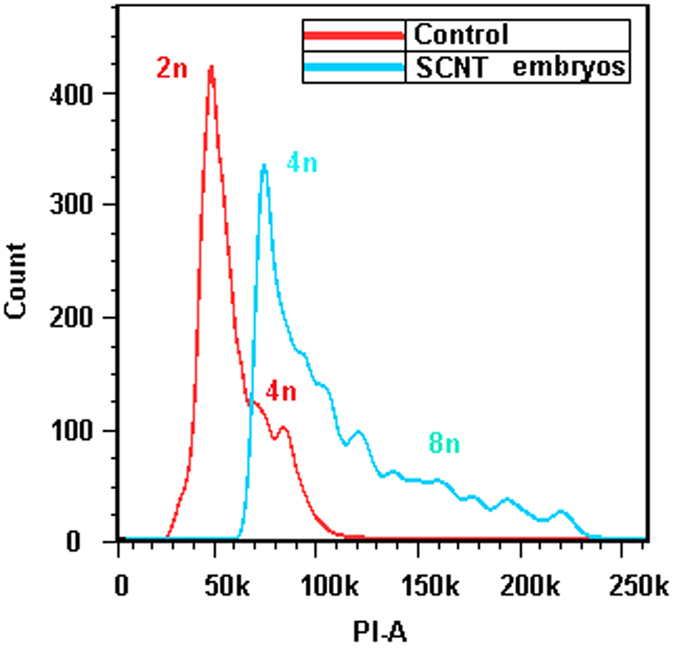
FACS analysis the SCNT embryos from the SP600125-induced tetraploid cells. (**A**) shows DNA content of the SCNT gastrula from the SP600125-induced tetraploid cells (blue) , and that of the gastrula of diploid crucian carp, which are used as the control (red).

We have repeated six times experiments of nuclear transfer with SP600125-induced autotetraploid cells. The results are shown in Table 1 and Fig. 6. It is clear that all the unfertilized crucian carp eggs without SCNT died before the multicellular stage, whereas the reconstructed embryos from the SP600125-induced autotetraploid cell nuclei and crucian carp eggs could develop forward. Specifically, we successfully operated on 922 reconstructed embryos. Among them, 420 embryos (45.56%) developed to blastula stage (6 h), and 73 embryos (7.91%) developed to gastrula stage (10 h). Though 55 SCNT gastrula (10 ~ 14 h) were selected from the gastrula embryos for next ploidy detection, there are still 18 SCNT embryos in the rest ones developed to neurula stage. Consequently, we obtained a larva of 48 h, which possesses blood circulation, muscular reaction and body pigment (Fig. 6). Data analysis by FACS indicates that the SCNT embryos randomly selected from the SCNT gastrula were tetraploid (Fig. 7). It suggests that the nuclei of SP600125-induced autotetraploid cells can be reprogrammed in the unfertilized eggs of crucian carp , and reversed to the totipluripotent state.

## Discussion

Polyploidization is important to evolution and generation of polyploid species. The chemical compound-induced cell arrest is critical way for generation of artificial polyploid cells. In this paper, SP600125 has been used to induce polyploidization of fish cells *in vitro* such that a stable fish tetraploid cell line has been obtained. We think that the presented method in this paper may be applicable to the polyploidization of other fish species, such as the economic fish.

Polyploidization may occur owing to abnormal cell division, usually during either mitosis or metaphase I in meiosis. The genetic stability of polyploid depends on the rapid restructure of genome and the changes in gene regulation^3^. SP600125 is a special Jnk inhibitor^17^,^19^,^20^. In our research, it is further shown that SP600125-induced polyploidization has no obvious impact on the activation of Jnk. Actually, knockdown of the *jnk* gene in diploid carp cells did not give rise to cell polyploidization. Thus, SP600125-induced polyploidization might occur by inhibiting other signal pathways, instead of Jnk one.

From the obtained results, it follows that some factors being related with cell cycle are involved in polyploidization. Cyclin dependent kinases (CDKs) are the key cell cycle regulators^11^,^21^. The CDK inhibitor, p21, has been reported to have different expression levels in normal diploid and non-diploid cells (such as cancer cells) as the downstream of the p53 signal pathway^12^,^21^. Our study reveals that both p21 and p53 expressions are up-regulated in the SP600125-induced tetraploid cells, especially compared with the diploid cells. Therefore, the p53 signal pathway might be important for maintaining the genetic stability. Actually, the existing results reported that the p53 signal pathway might regulate the nucleotide-excision repair of DNA, chromosomal recombination, and chromosome segregation^21^. No matter what, the SP600125-induced polyploidization mechanism needs further investigation in the future.

Although enucleated eggs were often used as recipient in the traditional nuclear transplant, non-enucleated eggs have also be applied to reconstruct the SCNT embryos by some researchers owing to its features of simplification and harmlessness in the reconstruction of embryos^22^,^23^. For example, in medaka, the diploid and fertile reconstructed fish have been successfully generated by transplanting the diploid nuclei into non-enucleated unfertilized eggs^23^,^24^. Furthermore, it was found that the SCNT fish with the recipient of non-enucleated diploidized eggs only has genetic characteristics of the donor, other than the recipient^25^. The reason might lie in that the nuclei of recipient eggs were eliminated^26^. More results also corroborated that the nuclei of recipient eggs were rejected and excluded in the SCNT embryos, especially in the case that the genetic background of the donor is close to that of the recipient^10^,^27^,^28^. In this article, by transplanting the nuclei of SP600125-induced autotetraploid cells into the non-enucleated unfertilized eggs of crucian carp, we have obtained the SCNT tetraploid embryos. To our knowledge, it is the first time to generate polyploidy by combination of polyploidization with the technique of somatic cell nuclear transfer. The results further demonstrate that non-enucleated unfertilized eggs can convert the donor nuclei into totipotent ones. Clearly, it may be a new approach to polyploid breeding.

With regard to reconstruction of the SCNT fish, low survival rates might be the main obstacles, either for medaka^22^,^29^, zebrafish^7^ or economic fish^10^,^30^. On the one hand, the lower developmental rate of the reconstructed embryos is caused by the uneven quality of recipient eggs and damage of surgical operation. On the other hand, the developmental rate of the reconstructed embryos is closely related with the developmental potentiality of donor cells. Actually, Siripattarapravat *et al.*^9^ found that the developmental rate of the SCNT embryos from adult caudal fin donor cells was far poorer than that from embryonic tail-bud donor cells. In mammal, aberrant epigenetic status was also found in the cloned embryos^31^ or cloned animals^32^. This study has reported that high deformity rate occurred in the reconstructed tetraploid embryos and fry, and only 7.91% of the SCNT embryos can develop to gastrula. Thus, to ensure higher survival rate of the SCNT tetraploid fish, one of the important directions in the future research should focus on exploration of molecular mechanism, which regulates the process of tetraploid nuclear reprogramming.

In summary, combination of polyploidization and SCNT is an efficient way to generate polyploidy, and the presented method in this research for generating the tetraploid fish from diploid fish is a useful platform for polyploid breeding.

## Materials and Methods

### Ethics Statement

This study was approved by the Animal Ethical Review Committee (AERC) of Hunan Normal University, Changsha, China. The collection of fish samples was permitted by the Engineering Center of Fish Breeding of the Education Ministry, Hunan Normal University. All sampling procedures were conducted according to the standards and ethical guidelines established by the AERC. And the researchers involved in animal experiment were certified under a professional training course for laboratory animal practitioners held by the Institute of Experimental Animals, Hunan Province, China.

### Cell Culture

Crucian carp (*Carassius auratus,* L.), 1 year old, were collected from the Engineering Center for Fish Breeding of the National Education Ministry, Hunan Normal University. Fish was anesthetized with 100 mg/L MS-222 (Sigma-Aldrich) before dissection. For primary cell culture *in vitro*, pieces of fin (~0.2 cm^2^) were obtained from the tail fin of crucian carp, then washed with PBS after a quick rinse with 70% alcohol. They were then digested with 0.25% trypsin (Invitrogen, Carlsbad, CA) for 15 ~ 30 min. Cells were cultured in the complete growth medium, which was composed of Dulbecco’s modified Eagles medium (DMEM; Sigma, St. Louis, MO) supplemented with 100 U/ml penicillin, 100 μg/ml streptomycin (Invitrogen, Carlsbad, CA), 10%fetal bovine serum (FBS, Invitrogen, Carlsbad, CA), 0.1% 2-mercaptoethan (2-ME, Invitrogen, Carlsbad, CA),1 mM sodium pyruvate (Invitrogen, Carlsbad, CA), and 1 mM nonessential amino acids (Invitrogen, Carlsbad, CA). Cells were grown in a 5% (v/v) CO_2_ at 28 °C, passaged every 2 or 3 days by trypsin. For cryopreservation, 10% dimethyl sulfoxide (DMSO; Sigma, St. Louis, MO) and 90% FBS were used.

### Tetraploid Cell Line Induction

100 μm SP600125 (C_14_H_8_N_2_O, Merk, Germany) was added to the complete growth medium when the diploid cells reached 80 ~ 90% confluence. 10%DMSO was used as the solvent for SP600125, and served as a negative control. 0.2 μg/L Colchicine (Sigma, St. Louis, MO), which can induce TIG-1 human fibroblast polyploidization was also used^4^,^5^, and served as a positive control.

### Purification of Tetraploid Cell Line by Fluorescence Activated Cell Sorting (FACS)

SP600125-induced cells were first digested into a single-cell solution and filtered through a 40 μm cell strainer. The cells were then incubated with 2 μg/ml Hoechst 33342 (Invitrogen, Carlsbad, CA) and 50 μM Verapamil (Sigma, St. Louis, MO) for 15 min at 28 °C and sorted in a Beckman MoFol XDP flow cytometer (Beckman-Coulter, USA). The tetraploid (4n) and octoploid (8n) peaks were collected. Purification was performed every five passages and recorded as one round. A stable tetraploid cell line was generated after two or more round. FACS data were analyzed by the ModFit software (Verity Software House, USA).

### Cell Growth Assays

Cell growth assays were performed with Thiazolyl blue tetrazolium bromide (MTT) kits following the manufacturer’s instructions (Sigma, St. Louis, MO). We seeded 1 × 10^4^ cells per well in 96-well plates and cultured them with 100 μl complete growth medium. After culturing for 24, 48, or 72 h at 28 °C, 10 μl MTT reagent was added to each well for 4 h until a purple precipitate was visible. The culture medium was changed to 100 μl DMSO to dissolve the formazan, and then cultured for 2 h in the dark. The absorbance at 570 nm was detected by a multilabel plate reader (Victor™X3, PerkinElmer, USA).

### Immunofluorescence

The cells were fixed in 4% paraformaldehyde for 30 min at 25 °C and blocked for 1 h with 2% BSA in PBS. Primary antibodies of the following markers were used: Tubulin (1:100, Millipore, Bedford, MA), p21 antibody (1:100, Abcam, Cambridge, MA), p53 antibody (1:100, Abcam, Cambridge, MA). Fluorescently labeled secondary antibodies were purchased from Jackson Lab (Sacramento, CA). DNA was stained with Hoechst 33342. Fluorescence was imaged using Zeiss LSM510 confocal microscope (Carl Zeiss AG, Oberkochen, Germany).

### Cell Nucleus Assays

The cells were seeded 0.8 × 10^4^ per well in 96-well plates and cultured in the complete growth medium at 28 °C. They were then fixed and stained with 1 μg/ml Hoechst 33342 for 15 min at 24, 48, and 72 h. Operetta High Content Screening (HCS) imaging system (PerkinElmer, CA) was used for the cell nucleus assays.

### Karyotyping

The confluent cells were treated with 0.2 μg/ml colchicine for 4 ~ 6 h, and then treated with a hypotonic solution of 0.075 M KCl for 30 min. The cells were fixed twice with cold Carnoy’s fixative (methanol/glacial acetic acid, 3:1, vol/vol), for 15 min each time, dropped on the slide, then air-dried. The chromosomes were stained with 5% Giemsa solution for 20 min. More than 30 metaphases were examined for each cell line.

### RNA Interference

*Jnk* shRNA lentiviral vector plasmids (shRNA *jnk1* Plasmid (m): sc-29381-SH; and shRNA *jnk3* Plasmid (m): sc-39104) was purchased from Santa Cruz (UK) and the three shRNAs were pooled. All of the plasmids contain a puromycin resistance gene. The RNAi experiment was carried out according to the manufacturer’s instructions. The primers used to test *jnk* knock-down were as the follows:

Sh*jnk1* forward: 5′-CAGCACCCCTACATCAACGT-3′

Sh*jnk1* reverse: 5′-TATCAGCTCTTTCCACTCCTC-3′

Sh*jnk3* forward: 5′-TGTCTAAGATGTTAGTGATTGA-3′

Sh*jnk3* reverse: 5′-ATCATATATCTGAGGCGGAGGC-3′

*β-actin* forward: 5′-CCTGGGTATGGAATCTTGCGG-3′

*β-actin* reverse: 5′-CGGTCAGCAATGCCAGGGTA3′

### Western Blotting

The cells were lysed with lysis buffer supplemented with a protease inhibitor cocktail (Merck, Germany). The protein concentration was measured with a Bradford assay kit (Bio-Rad, USA). Fifty-μg of cell lysate was resolved on a 10% SDS-polyacrylamide gel and transferred to a polyvinylidine difluoride membrane (Millipore, Bedford, MA). The membrane was blocked with 5% skim milk dissolved in PBS containing 0.1% Tween20 (0.1% PBST). After blocking, the blot was incubated with primary antibodies (anti-JNK (Cell signaling), anti-P-JNK (Cell signaling), anti-GAPDH (Cell signaling)) overnight at 4 °C with gentle shaking. After washing with 0.1% PBST three times, secondary antibodies were added for 60 min. The signals were detected by SuperSignal^®^ West Pico Chemiluminescent Substrate (Thermo, USA).

### Preparation of Recipient Eggs and Donor cells for nuclear transfer

Unfertilized eggs were from crucian carp, and placed into a trypsin solution of 0.25% (w/v; Sigma) for 1 min, and the softened chorions were subsequently removed by microsurgery. Eggs were held in a 1.5% agar plate filled with Danien’s buffer solution (3.390 g/L NaCl, 0.052 g/L KCl, 0.237 g/L HEPES, 0.142 g/L Ca(NO_3_)_2_.4H_2_O, 0.099 g/L MgSO_4_.7H_2_O, 0.250 g/L NaHCO_3_) for further manipulation. Prior to nuclear transfer, strep to mycin (100 U/ml) and ampicillin (100 U/ml) were added to the working medium and mixed briefly.

The SP600125-induced autotetraploid cells at the 26-th passage were digested by 0.25% trypsin and centrifuged at 800 rpm for 5 min. Then, they were pipetted in phosphate balanced solution (PBS), centrifuged at 800 rpm for 5 min, and resuspended in the solution of culture medium without FBS.

### Nuclear Transplantation

Unfertilized crucian carp eggs without chorions were maintained in a 1.5% agar (w/v; Sigma) plate filled with Danien’s solution. Nuclear transfer was conducted using either by an Eppendorf microinjection system (Model 5171/5246, Hamburg, Germany) with a Nikon TE300 microscope (Nikon, Melville, NY, USA) or by a Narishige system (NT-188NE, Leeds Precision Instruments, Minneapolis, MN, USA) with an Axiovert 200 microscope (Carl Zeiss). Donor cells were ruptured by aspiration into the transfer needle, whose inner diameter is 12-μm or so, smaller than the cell. Then, they were transplanted into the cytoplasm of the eggs at the animal pole. The SCNT embryos were transferred into an agar plate filled with Danien’s solution. The unfertilized crucian carp eggs without chorions were served as the negative control. The SCNT embryos and negative control eggs were cultured in Danien’s solution at 28 °C, respectively.

### Ploidy analysis of the SCNT embryos

The SCNT embryos at gastrula stage (10 ~ 14 h) were first randomly selected. Then, they were digested into a single-cell solution, filtered through a 40 μm cell strainer, and incubated with 2 μg/ml Hoechst 33342 (Invitrogen, Carlsbad, CA) and 50 μM Verapamil (Sigma, St. Louis, MO) for 15 min at 28 °C. Finally, they were sorted in a Beckman MoFol XDP flow cytometer (Beckman-Coulter, USA). The obtained data from FACS were analyzed by the ModFit software (Verity Software House, USA). The gastrula stage embryos of diploid crucian carp were served as the control.

## Additional Information

**How to cite this article**: Zhou, Y. *et al.* Autotetraploid cell Line induced by SP600125 from crucian carp and its developmental potentiality. *Sci. Rep.* **6**, 21814; doi: 10.1038/srep21814 (2016).

## Supplementary Material

Supplementary Information

## Figures and Tables

**Table 1:** Development of cloned embryos.

Groups	No. Of Egg operated	Blastula		Gastrula	
		Number	%	Number	%
Exp.1	100	60	60.0%	27	27.0%
Exp.2	110	53	48.2%	11	10.0%
Exp.3	133	59	44.4%	14	10.5%
Exp.4	183	52	28.4%	7	3.8%
Exp.5	216	129	59.7%	6	2.7%
Exp.6	180	67	37.2%	8	4.4%
Control	922	0	0.0%		

Note: Exp.1 to Exp.6 denote the repeated six times experiments of nuclear transfer with SP600125-induced autotetraploid cells, and Control is the result of the unfertilized crucian carp eggs without SCNT corresponding to each experiment.
