# Supplementary material for: Autotetraploid cell Line induced by SP600125 from crucian carp and its developmental potentiality
Source: Sci Rep. 2016 Feb 22;6:21814. doi: 10.1038/srep21814 (PMC4761888; doi:10.1038/srep21814)
Supplement: Supplementary Information [file srep21814-s1.pdf]

Supplementary Information

Autotetraploid cell Line induced by SP600125 from crucian carp and its developmental potentiality

Yonghua Zhou[1¶], Mei Wang[1,2¶], Minggui Jiang[1,2], Liangyue Peng[1], Cong Wan[1], Jinhui Liu[1], Wenbin Liu[1], Rurong Zhao[1], Xiaoyang Zhao[2], Wei Hu[3], Shaojun Liu[1] and Yamei Xiao[1*]

[1]Key Lab of Protein Chemistry and Developmental Biology of Education Ministry of China, College of Life Sciences, Hunan Normal University, Changsha, 410081, China

[2]State Key Laboratory of Reproductive Biology, Institute of Zoology, Chinese Academy of Sciences, Beijing, 100101, China

[3] The National Key Laboratory of Freshwater Ecology and Biotechnology, Institute of Hydrobiology, Chinese Academy of Sciences, Wuhan, 430072, China

[¶]These authors contributed equally to this work.

*Corresponding author

E-mail: yameix@126.com

Running title: Generation of tetraploid cell line from diploid crucian carp

Supplementary Fig. S1

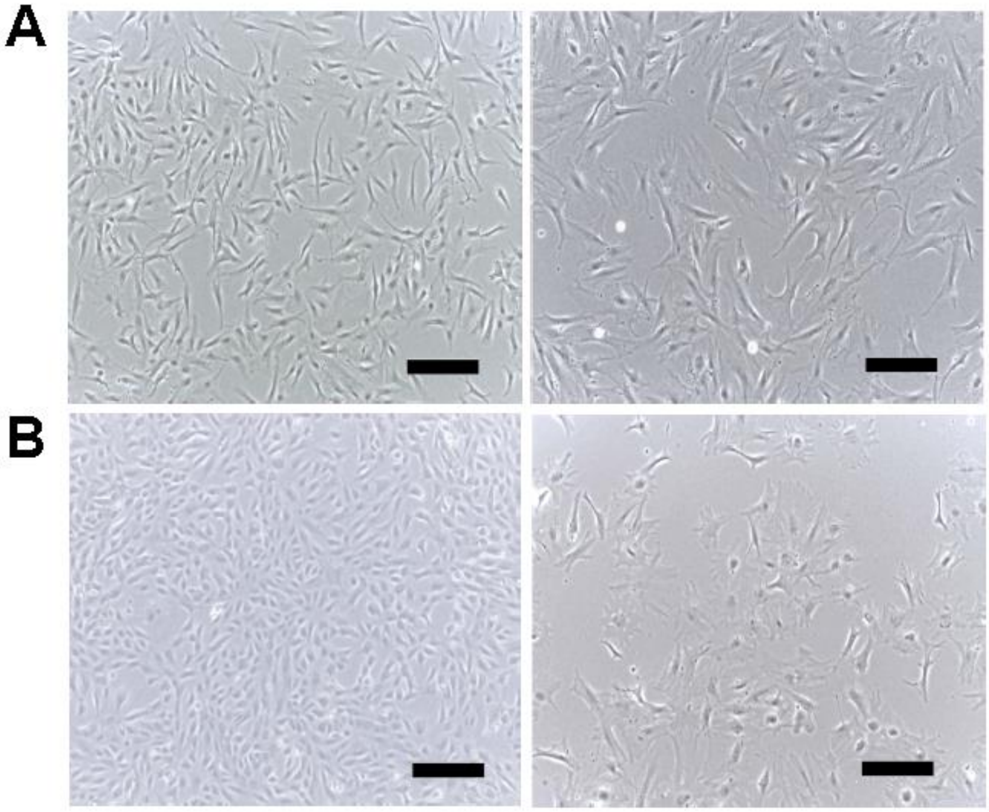

Supplementary Fig. S1. Morphology of cells treated with SP600125

(A). Morphology of the crucian carp cells treated with DMSO (left，as control) and SP600125 (right) for 48h. Scale bars represent 200μm.

(B). Morphology of the collected 2n cells treated with DMSO (left, as control) and the collected 4n crucian carp cells treated with SP600125 (right) for another72h, Scale bars represent 200 μm.

Supplementary Fig. S2

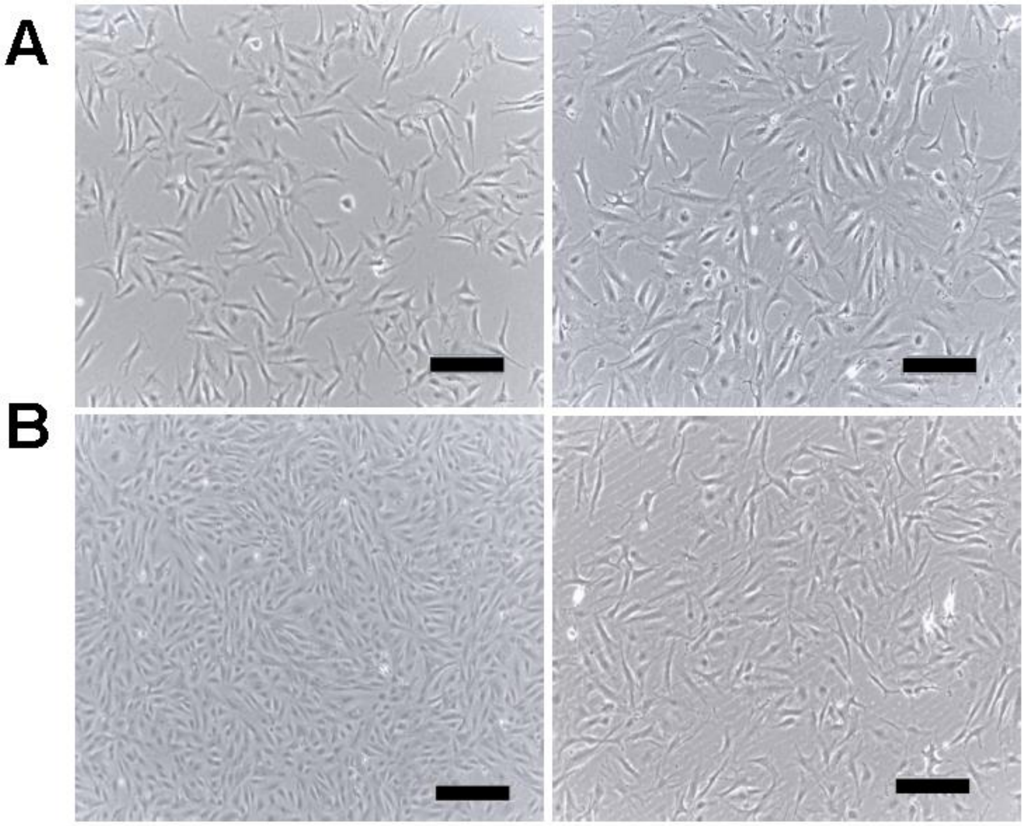

Supplementary Fig. S2. Morphology of cells of tetraploid crucian carp cell line

(A). Morphology of the FACS collected 2n peak cells (left), 4n and 8n peak cells (right) were cultured again in SP600125-free medium for 12h, respectively. Scale bars represent 200μm;

(B). Morphology of the crucian carp cells before sorted that within 5 cycles of DMSO（left）, and SP600125 (right) treated in five passages, respectively. Scale bars represent 200μm;

Supplementary Fig. S3

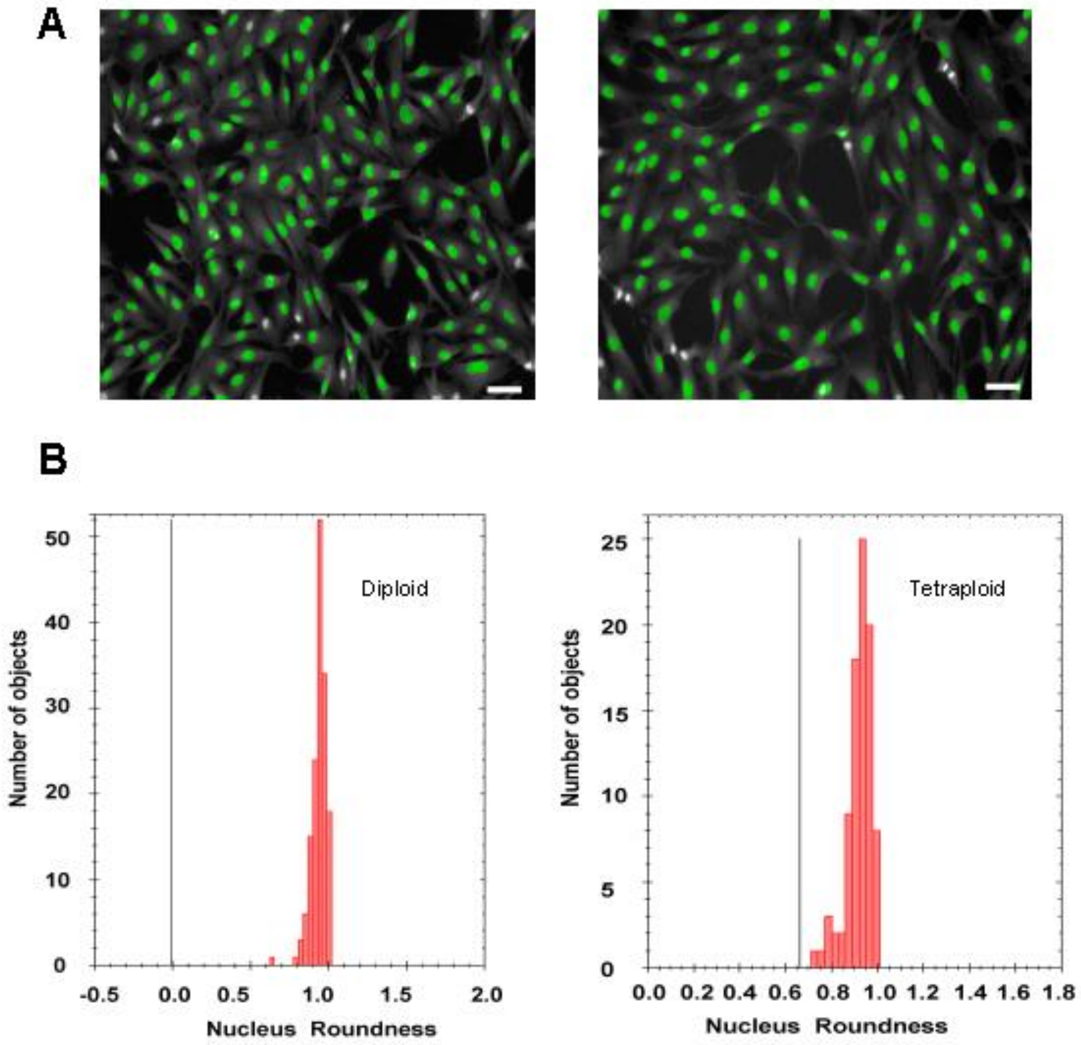

Supplementary Fig. S3. Characteristics of SP600125-inducted tetraploid cells tested by high content assays

(A). Morphology of the diploid cells (left) and tetraploid (right) tested by high content assays, respectively. Scale bars represent 100μm;

(B).The nucleus roundness of diploid cells (left) and tetraploid (right) detected by high content assays, respectively.

Supplementary Fig. S4

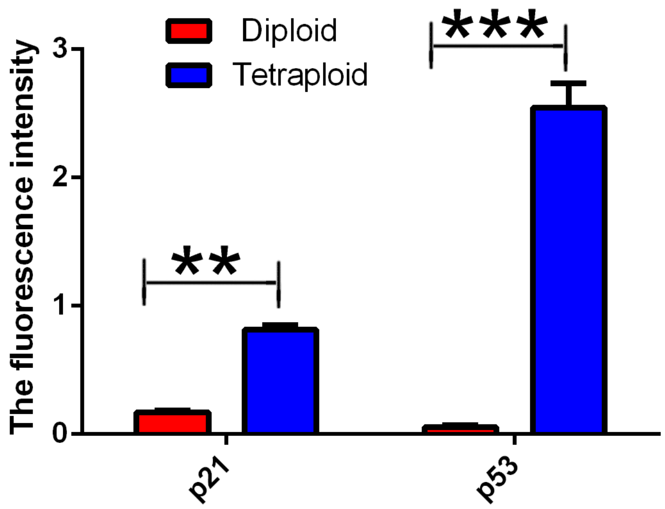

Supplementary Fig. S4. The fluorescence intensity p21 and p53 of the diploid cells (red) and tetraploid (blue) tested by Image J. Error bars represent the means ±SD; n=10. P<0.001.
